# AMD3100 reduces CXCR4-mediated survival and metastasis of osteosarcoma by inhibiting JNK and Akt, but not p38 or Erk1/2, pathways in *in vitro* and mouse experiments

**DOI:** 10.3892/or.2015.3992

**Published:** 2015-05-19

**Authors:** YU-XIN LIAO, ZE-ZE FU, CHENG-HAO ZHOU, LIAN-CHENG SHAN, ZHUO-YING WANG, FEI YIN, LONG-PO ZHENG, YING-QI HUA, ZHENG-DONG CAI

**Affiliations:** 1 Department of Orthopaedics, Shanghai Tenth People’s Hospital, Tongji University School of Medicine, Shanghai 200072, P.R. China; 2 Department of Orthopaedics, Shanghai First People’s Hospital, Shanghai Jiaotong University School of Medicine, Shanghai 200080, P.R. China; 3 Postdoctoral Research Station of School of Life Science and Technology of Tongji University, Shanghai 200092, P.R. China

**Keywords:** osteosarcoma, chemokine 12, chemokine receptor 4, AMD3100, survival, metastasis

## Abstract

Osteosarcoma (OS) has an unfavorable prognosis and tends to metastasize to lung tissue. Although the CXCL12-CXCR4 axis appears to affect progression and metastasis in numerous tumors, its mechanism and downstream pathways in OS remain unclear. We used western blotting and flow cytometry to detect CXCR4 and CXCR7 expression in two OS cell lines (LM8 and Dunn). An MTT assay was used to evaluate the effects of CXCL12 and AMD3100, a specific CXCR4 antagonist, on cell viability. Flow cytometry was utilized to analyze changes in apoptosis induced by serum deprivation following treatment with CXCL12 and AMD3100. A Transwell assay was used to assess cell migration in response to CXCL12 and AMD3100. Western blotting was performed to identify the phosphorylation of signaling molecules (JNK, c-Jun, Akt, p38 and Erk1/2) and expression of caspase-3 and -8, and PARP. Mouse models were employed to evaluate AMD3100 inhibition of primary OS growth and lung metastasis *in vivo*. CXCR4 expression was detected in LM8 but not Dunn cells, and neither cell line expressed CXCR7. The addition of CXCL12 induced the survival and migration of serum-starved CXCR4^+^ LM8 cells activating JNK and Akt pathways, which were abrogated by adding AMD3100. However, similar results were not observed in CXCR4^−^ Dunn cells. CXCL12 protected LM8, but not Dunn cells, from apoptosis induced by serum deprivation by suppressing PARP cleavage, which was partly reversed by AMD3100. In a mouse model, AMD3100 reduced primary tumor growth and lung metastasis compared with the controls. Thus, the CXCL12-CXCR4 axis regulated OS survival and metastasis through the JNK and Akt pathways, and blocking them with AMD3100 was found to be a potential OS treatment.

## Introduction

Osteosarcoma (OS) is the most common primary bone malignancy. It mainly affects children and adolescents (1), and is often localized to the distal femur and proximal tibia. OS has a high propensity for lung metastasis, which is the leading cause of OS-related mortality, with 13–27% of OS patients presenting with lung metastases at diagnosis and 40% eventually developing metastasis (2–4). Despite advances in adjuvant chemotherapy and surgical resection, 5-year survival rates for OS patients without and with metastases are 60–65 and 20–29%, respectively (4). To better understand this disease, the mechanisms that regulate its progression and metastasis must be elucidated.

Chemokines and their receptors were initially identified as mediators of leukocyte migration to infection and inflammation sites, but have been increasingly shown to modulate tumor development, angiogenesis and metastasis (5–7). Chemokines are a superfamily of 8- to 12-kDa chemoattractant cytokines that are constitutively secreted by stromal cells, including fibroblasts and endothelial cells (8,9). Chemokine receptors are G protein-coupled seven-transmembrane cell-surface receptors to which their ligands bind with high affinity (10). At present, more than 50 chemokines and at least 20 chemokine receptors have been confirmed. Chemokines and their receptors are divided into four groups: C, CC, CXC, CX3C for chemokines and CXCR, CCR, XCR, CX3CR for its receptors based on the number and position of conserved cysteines, where C is the number of cysteine residues and X denotes the number of amino acids between conserved cysteines (10). The CXC subgroup is further classified into ELR^+^ and ELR^−^ chemokines, based on the presence of the tripeptide glutamic acid-leucine-arginine (the ‘ELR’ motif) (11). Notably, ELR^+^ CXC chemokines stimulate angiogenesis, whereas most ELR^−^ CXC chemokines inhibit angiogenesis, with the exception of CXCL12, an angiogenic ELR^−^ CXC chemokine (11).

Of all the chemokines and their receptors, the interaction between CXCL12 and its receptor CXCR4 is the most widely studied due to its pivotal role in carcinogenesis (12). CXCL12, also designated as stromal cell-derived factor-1 (SDF-1), is secreted by stromal cells including fibroblasts and endothelial cells, and is widely expressed in many organs including lung, liver, skeletal muscle, brain, kidney, heart, skin and bone marrow (8). CXCR4 was initially identified as the co-receptor that facilitates entry of T-tropic (X4) HIV viruses into CD4^+^ T cells. CXCR4 is the cognate receptor for CXCL12 (13,14), and its expression has been found in a wide range of tissues including brain, lymph node and small intestine (15) and monocytes, B and naive T cells, and early hematopoietic progenitor cells in the immune system (16). More importantly, CXCR4 overexpression has been detected in at least 23 different types of human cancer (17). As tumor cells that express CXCR4 are thought to be more likely to migrate to organs with abundant sources of CXCL12 (10), CXCR4 has long been considered a crucial mediator of metastasis for various types of tumors including OS (9,18–22). Hypoxia promotes human OS migration by inducing CXCR4 expression via hypoxia-inducible factor-1α (HIF-1α) activation (23). The anti-metastatic effect of sorafenib chemotherapy on human OS cells includes CXCR4 downregulation (24). Lung metastases of human OS cell-derived orthotopic xenografts in a mouse model could be suppressed by CXCR4 antibody treatment (25). However, to the best of our knowledge, few studies have shown that the expression of CXCL12 and CXCR4 correlates with a better long-term outcome and with a lower prevalence of metastases in OS (26). Despite accumulating evidence that CXCL12 and CXCR4 affect proliferation and growth of ovarian (27), prostate (28,29) and breast cancer (9,30,31), glioma (32), glioblastoma (33,34), esophageal cancer (35), Ewing’s sarcoma (36) and chondrosarcoma (7), the role of the CXCL12-CXCR4 axis in the survival and growth of OS cells remains to be investigated. Miura *et al* (37) showed that the ability to form tumors *in vivo* positively correlated with CXCR4 levels in human OS cells.

The CXCR4 antagonist, AMD3100, is a small bicyclam molecule that was originally used to prevent X4-Tropic HIV-1 viruses entering into CD4^+^ T cells via CXCR4 (38). It was subsequently approved to treat multiple myeloma and lymphoma due to its safety and efficiency in stimulating hematopoietic stem cell mobilization (39,40). CXCR4 inhibition from AMD3100 reportedly decreases the CXCL12-induced migration of OS cells (22,41). However, little is known about the effect of AMD3100 on OS cell survival and growth, or the exact mechanisms of CXCL12-CXCR4 interaction and the effect of AMD3100 on downstream pathways. In recent years, more attention has been paid to the participation of CXCR7, a novel decoy receptor of CXCL12, in the CXCL12-CXCR4-mediated OS progression and metastasis. The critical role of CXCR7 in mediating OS progression in the lungs and its lung metastasis-enhancing effect on OS expressing CXCR4 has been reported (42,43). CXCR7 is also found to be involved in OS proliferation (44).

In the present study, we aimed to: i) detect the expression of CXCR4 and CXCR7 in two OS cell lines; ii) investigate the roles of the CXCL12-CXCR4 axis and AMD3100 in OS cell survival and migration *in vitro*; iii) identify downstream pathways regulated by CXCL12/CXCR4 in OS; and iv) assess the effect of AMD3100 on primary tumor growth and lung metastasis in an OS animal model.

## Materials and methods

### Cell culture and reagents

The murine LM8 and Dunn OS cell lines were kindly donated by Dr Eugenie Kleinerman (M.D. Anderson Cancer Center, Houston, TX, USA). The cell lines were cultured in high-glucose Dulbecco’s modified eagle’s medium (DMEM-h; Thermo Fisher Scientific, Waltham, MA, USA) supplemented with 10% fetal bovine serum (FBS), 100 U/ml penicillin and 100 *μ*g/ml streptomycin. The cultures were incubated at 37°C in a humidified atmosphere containing 5% CO_2_. Recombinant murine CXCL12 was purchased from PeproTech (Rocky Hill, NJ, USA) and AMD3100 from Merck (Darmstadt, Germany). The rabbit antibodies (Ab) used for western blotting were obtained from different resources: anti-CXCR4 polyclonal Ab, anti-CXCR7 polyclonal Ab (both from Abcam, Cambridge, MA USA), anti-phospho-SAPK/JNK monoclonal Ab (MAb) (Thr183/Tyr185), anti-SAPK/JNK MAb, anti-phospho-c-Jun MAb (Ser73), anti-c-Jun MAb, anti-phospho-Akt MAb (Ser473), anti-Akt MAb, anti-phospho-p38 MAPK MAb (Thr180/Tyr182), anti-p38 MAPK MAb, anti-phospho-p44/42 MAPK (Erk1/2; Thr202/Tyr204) MAb, anti-p44/42 MAPK (Erk1/2), anti-caspase-3 MAb, anti-caspase-8 MAb and anti-PARP MAb (all from Cell Signaling Technology, Inc., Danvers, MA, USA), and anti-β-actin (Santa Cruz Biotechnology, inc. Santa Cruz, CA, USA).

### MTT assay

The effects of CXCL12 and AMD3100 on the survival of two OS cell lines (LM8 and Dunn) were assessed using a 3-(4,5-dimethylthiazol-2-yl)-2,5-diphenyltetrazolium bromide (MTT) assay. Cells were seeded in 96-well plates at 2×10^3^/well in DMEM-h. After overnight growth, the cells were cultured for 7 days in FBS-free medium in the presence of 0 or 100 ng/ml CXCL12, 30 *μ*M AMD3100 alone or 100 ng/ml CXCL12 with 10, 20 or 30 *μ*M AMD3100. The FBS-free cells without 100 ng/ml CXCL12 or AMD3100 served as the control group. After the 7 day incubation, 20 *μ*l MTT (5 mg/ml; Sigma, St. Louis, MO, USA) was added into each well and incubated for 4 h at 37°C. Culture medium was removed and 150 *μ*l dimethylsulfoxide was added. The optical density (OD) was then measured using a model ELx800 microplate reader (Bio-Tech instruments inc.) at 490 nm. The cell viability was calculated using the equation: Cell viability (%) = (OD^490nm^ of treatment/OD^490nm^ of control) ×100%.

### Migration assay

The migration assay was performed using Transwell assay (Corning Inc., Corning, NY, USA; pore size, 8-*μ*m) in 24-well dishes. Prior to the assay, OS cells (LM8 and Dunn) were pretreated for 24 h in FBS-free medium with or without 30 *μ*M AMD3100. Then, 1×10^5^ cells in 200 *μ*l of FBS-free DMeM-h medium were placed in the upper chamber, and 500 *μ*l of the same medium containing 100 ng/ml CXCL12 in the lower chamber. Additionally, 500 *μ*l FBS-free and 10% FBS DMeM-h medium was placed in the lower chamber as negative and positive controls, respectively. Plates were incubated for 48 h at 37°C in 5% CO_2_; culture medium was then removed and filters were washed with phosphate-buffered saline (PBS) twice. The cells on the upper side of filters were removed with cotton-tipped swabs. The cells on the underside of the filters were fixed in 95% alcohol for 10 min and stained with 0.1% crystal violet for 15 min. Cells that migrated from the upper to the lower side of the filter were counted under a light microscope by counting 10 random fields at a magnification of ×100. This assay was performed in triplicate.

### Flow cytometric analysis

To detect CXCR4, LM8 and Dunn cells (3×10^5^/well) were incubated in 6-well plates with DMEM-h + 10% FBS for 24 h, washed in cold PBS three times, and centrifuged at 1000 rpm for 5 min and resuspended them in 50 *μ*l PBS in flow cytometry (FCM) tubes. The cells were incubated with 5 *μ*l phycoerythrin (PE)-conjugated rabbit anti-mouse CXCR4 or PE-conjugated rabbit anti-mouse IgG2b (κ as an isotype control) at 4°C for 45 min, and analyzed by FCM using FACSCalibur and CellQuest software (both from BD Biosciences, San Jose, CA, USA).

To observe the effects of CXCL12 and AMD3100 on cell apoptosis, LM8 and Dunn cells (1×10^5^/well) were cultured in FBS-free medium for 7 days with 0 or 100 ng/ml CXCL12, or 100 ng/ml CXCL12 with 10, 20 or 30 *μ*M AMD3100. After the 7 day incubation, the cells were washed twice in cold PBS, centrifuged and mixed in 100 *μ*l 1Χ binding buffer, and incubated in the dark at room temperature for 15 min with 5 *μ*l of Annexin V-FiTC and 5 *μ*l of propidium iodide (Pi) prior to analysis. Apoptosis was detected by FCM and analyzed using CellQuest software. Dual parameter dot plots combining Annexin V-FITC/PI showed living cells in the lower-left (Annexin V^−^/PI^−^), early apoptotic cells in the lower right (Annexin V^+^/PI^−^), late apoptotic cells in the upper right (Annexin V^−^/PI^+^), and necrotic cells in the upper left (Annexin V^+^/PI^+^) quadrant. Total apoptotic rates were calculated as sums of rates observed in the lower- and upper-right quadrants (45).

### Western blot analysis

Western blotting was used to detect CXCR4 and CXCR7 in LM8 and Dunn cells, and phosphorylation of signaling molecules. The cells were starved in FBS-free medium for 48 h in the presence of 0 or 100 ng/ml CXCL12, or 100 ng/ml CXCL12 with 10, 20 or 30 *μ*M AMD3100. Cellular lysates were obtained using RiPA lysis buffer (Beyotime institute of Biotechnology, Shanghai, China), protease and phosphatase inhibitors (Thermo Scientific). Protein concentrations were calculated using the Pierce BCA protein assay kit (Thermo Scientific). Equivalent amounts of total protein (30 *μ*g) from each sample were separated by electrophoresis on 10% SDS-polyacrylamide gels at 80 volts and transferred to nitrocel lulose membranes. After blocking with TBST containing 5% skim milk for 1 h, the membranes were incubated overnight at 4°C with 1:1,000 dilution of rabbit primary antibodies [CXCR4, CXCR7, phospho-SAPK/JNK, SAPK/JNK, phospho-c-Jun, c-Jun, phospho-Akt, Akt, phospho-p38 MAPK, p38 MAPK, phospho-p44/42 MAPK (Erk1/2), p44/42 MAPK (Erk1/2), caspase-3 and -8, PARP, β-actin], followed by incubation with secondary antibodies (goat anti-rabbit IgG) at room temperature for 1 h. Expression of proteins was analyzed using the Odyssey infrared laser imaging system.

### Animal experiments

Ten 4-week-old female C3H mice were purchased from the Chinese Academy of Sciences (Shanghai, China). The mice were housed under standard conditions with a 12-h light-dark cycle and fed with sufficient water and food. All the animal procedures were performed in accordance with a protocol approved by the Animal Care and Use Committee of Shanghai Tongji University. A single-cell suspension of ~10^6^ LM8 cells in 10 *μ*l of PBS was injected into the right tibia medullary cavity to establish an orthotopic animal model of OS. Two weeks after transplantation, the mice were randomly allocated to the AMD3100-treated (n=6) and the PBS-treated group (n=4; controls). Each mouse in the treatment group received 100 *μ*l AMD3100 (5 mg/kg, diluted with PBS) by tail vein injection every 2 days. The control mice were injected with 100 *μ*l PBS in the same manner. After 10 continuous injections in the two groups, the mice were euthanized to observe the primary tumor size. Posterior limbs with tumors were weighed. Lung metastasis was detected by hematoxylin and eosin (H&E) staining.

### Statistical analysis

All the experiments were repeated at least three times. Statistical analysis was performed with GraphPad Prism v5.0 (GraphPad Software, La Jolla, CA, USA). Measurement data are presented as mean ± standard deviation (SD). Experiments with two groups were analyzed using the Student’s t-test. P<0.05 was considered to indicate a statistically significant result.

## Results

### Expression of CXCR4 and CXCR7 in OS cells

**Figure 1:**
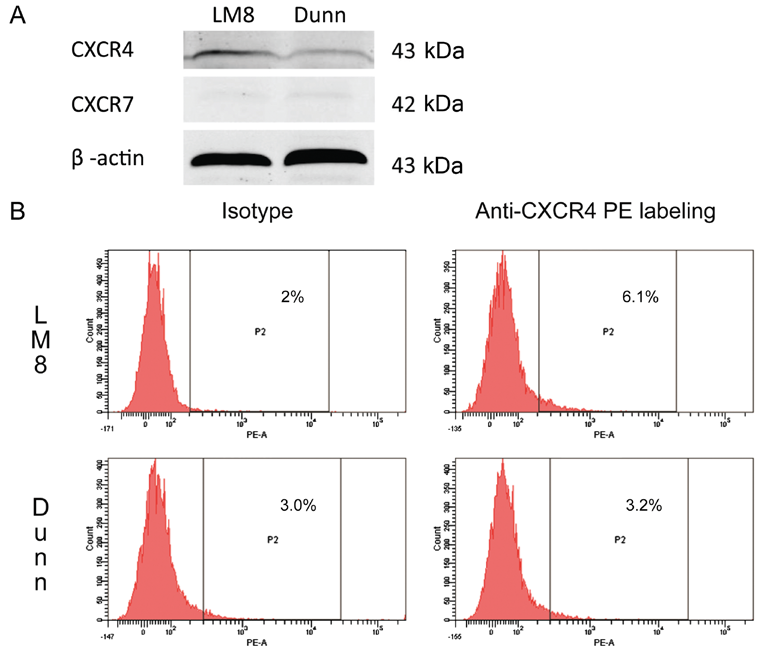
CXCR4 and CXCR7 expression in murine osteosarcoma cells. (A) CXCR4 and CXCR7 protein expression in LM8 and Dunn cells was detected by western blotting; 30 *μ*g of cellular protein were separated on a 10% SDS-polyacrylamide gel and transferred to nitrocellulose membranes incubated with CXCR4 and CXCR7 antibodies. (B) CXCR4 cell-surface expression was evaluated by flow cytometry. The cells that diverged from the control samples stained with isotype and PE anti-mouse CXCR4 were regarded as CXCR4^+^. PE, phycoerythrin.

Western blotting was used to determine whether CXCR4 and CXCR7 were expressed in LM8 and Dunn cells. LM8 but not Dunn cells, expressed high CXCR4 protein levels (Fig. 1A). Similarly, FCM showed that 4.1% of LM8 cells, but only 0.2% of Dunn cells, expressed cell-surface CXCR4 (Fig. 1B). The LM8 and Dunn cell lines did not express CXCR7 (Fig. 1A).

### CXCL12 enhances serum-starved OS cell survival by binding CXCR4, but this effect can be abrogated by AMD3100

**Figure 2:**
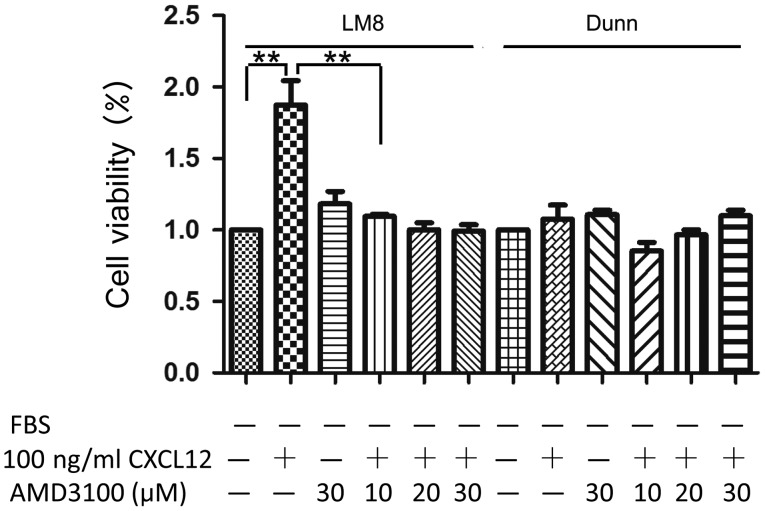
Effects of CXCL12 and AMD3100 on the survival of osteosarcoma cells *in vitro*. Cell viability was measured by an MTT assay. CXCL12 enhances survival of CXCR4^+^ LM8 cells serum-starved for 7 days. AMD3100-induced CXCR4 inhibition significantly decreased CXCL12-induced survival in LM8 cells, but not dose-dependently, suggesting the influence of other factors. Such results were not observed in Dunn cells. FBS, fetal bovine serum.

To investigate the effects of CXCL12 and AMD3100 on cell survival, OS cells were cultured for 7 days in FBS-free medium in the presence of 0 or 100 ng/ml CXCL12, 30 *μ*M AMD3100 alone, or 100 ng/ml CXCL12 with 10, 20 or 30 *μ*M AMD3100, and then analyzed for cell viability. The results showed that 100 ng/ml CXCL12 significantly increased the survival rate of CXCR4^+^ LM8 cells compared with the controls (P<0.05, Fig. 2). However, no similar effect was observed in CXCR4-Dunn cells (P>0.05, Fig. 2). As the two cell lines lacked CXCR7 expression (as described above), the function of which should be taken into account in the effects of CXCL12/CXCR4 on tumor survival, these results suggested that CXCL12 promotes OS cell survival through CXCR4 receptors in the absence of serum.

To confirm whether CXCL12-induced cell survival depends on binding to CXCR4, we used the CXCR4 antagonist AMD3100 (10, 20 or 30 *μ*M) to block CXCR4 in OS cells. We found that inhibition of CXCR4 by AMD3100 significantly reduced LM8 cell survival in the presence of CXCL12 (P<0.05, Fig. 2), but had no effect on CXCR4^−^ Dunn cells. However, the AMD3100 inhibition of LM8 cell survival was not dose-dependent (P>0.05, Fig. 2). Furthermore, survival of LM8 cells treated with 30 *μ*M AMD3100 alone was not altered (P>0.05, Fig. 2). These results showed that blocking CXCR4 with AMD3100 decreases the CXCL12-induced survival of CXCR4^+^ OS cells, and this effect was not concentration-dependent.

### CXCL12 protects serum-starved CXCR4^+^ OS cells from apoptosis and this effect can be reversed by AMD3100

**Figure 3:**
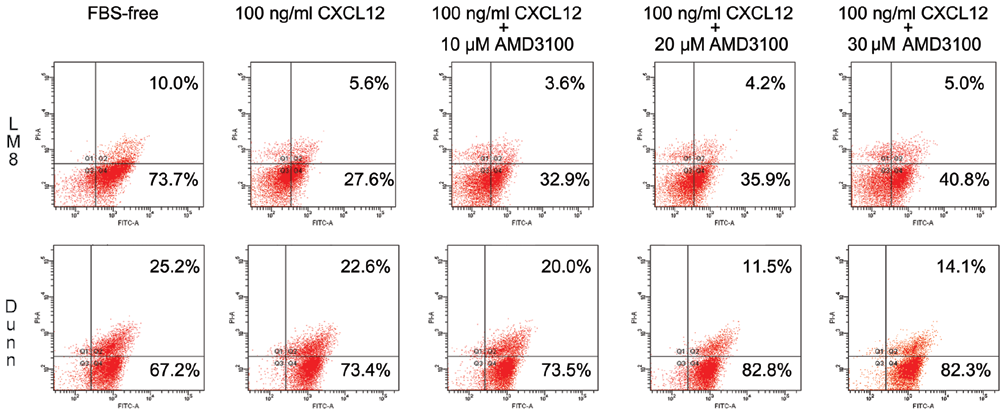
CXCL12 protects CXCR4^+^ LM8 cells from apoptosis induced by serum deprivation. Flow cytometric analysis showed apoptosis in serum-starved LM8 to be 33.2% in the presence of CXCL12, compared with 83.7% in the control group. However, CXCL12 had no similar effect on CXCR4^−^ Dunn cells. AMD3100 (30 *μ*M) partly reversed the anti-apoptosis effect of CXCL12 by inhibiting CXCR4. FBS, fetal bovine serum.

To examine whether CXCL12 and AMD3100 affect apoptosis, cells were starved in FBS-free medium for 7 days with 0 or 100 ng/ml CXCL12, or 100 ng/ml CXCL12 with 10, 20 or 30 *μ*M AMD3100. The total percentage of apoptotic LM8 cells (including early- and late-stage apoptosis) in the FBS-free group was 83.7%, but only 33.2% in the 100 ng/ml CXCL12 group (P<0.05), and 36.5, 40.1 or 45.8%, respectively, in LM8 cells treated with 100 ng/ml CXCL12 combined with 10, 20 or 30 *μ*M AMD3100 (Fig. 3). However, the percentages of apoptotic Dunn cells did not significantly vary between the FBS-free control group (92.4%) and the 100 ng/ml CXCL12 group (96.0%; P>0.05, Fig. 3), and were 93.5, 94.3 or 96.4%, respectively, in Dunn cells treated with different AMD3100 concentrations. These results showed that CXCL12 prevents apoptosis in serum-starved CXCR4^+^ OS cells via the activation of CXCR4. As blocking CXCR4 with 30 *μ*M AMD3100 led to significantly higher apoptosis percentages compared with the CXCL12 group (P<0.05), the addition of 30 *μ*M AMD3100 in CXCL12 appeared to partly reverse its anti-apoptotic effect.

### AMD3100 impairs CXCR4-dependent, CXCL12-induced migration of OS cells

**Figure 4:**
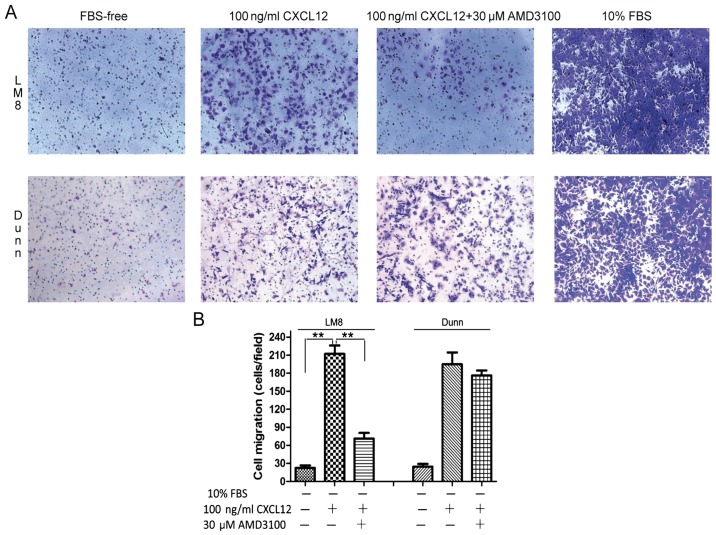
AMD3100 significantly reduces CXCR4-mediated, CXCL12-induced migration of LM8 cells. Osteosarcoma cell lines LM8 and Dunn in FBS-free medium were seeded in Transwell upper chambers and 100 ng/ml CXCL12 as a chemoattractant was placed in the lower chamber. After 48 h, the cells on the upper filter surfaces were removed and stained with crystal violet. (A) Representative staining; magnification, ×100. (B) Migrated cells were quantified by counting cells from 10 random fields at a magnification of ×100. FBS, fetal bovine serum.

To determine whether CXCL12 regulated OS migration, the cells were treated in FBS-free medium with 0 or 30 *μ*M AMD3100 for 24 h before adding CXCL12. The number of migrated LM8 cells increased by 83.7% in the presence of CXCL12 compared with the negative controls (Fig. 4A and B), whereas the migration of LM8 cells pretreated with AMD3100 was notably inhibited in response to CXCL12. Notably, CXCL12 also attracted migration of CXCR4^−^ Dunn cells (Fig. 4A and B), whereas AMD3100 pretreatment did not decrease their CXCL12-induced migration due to CXCR4 deficiency. These results suggested that CXCR4 inhibition by AMD3100 significantly reduced CXCR4-mediated, CXCL12-induced OS cell migration.

### CXCL12 activates JNK and Akt pathways in OS by binding to CXCR4

**Figure 5:**
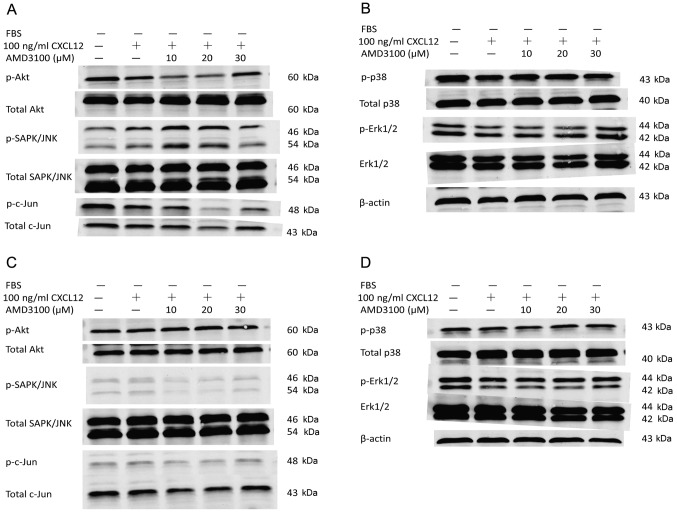
CXCL12 activates JNK and Akt pathways in LM8 cells by binding to CXCR4, which was inhibited by AMD3100. Cells were starved in FBS-free medium for 48 h with 0 or 100 ng/ml CXCL12, or 100 ng/ml CXCL12 with 10, 20 or 30 *μ*M AMD3100. Cells were then subjected to western blot analysis to detect phosphorylation of JNK, c-Jun, Akt, p38 and erk1/2. Phosphorylation of (A) JNK, c-Jun and Akt and (B) p38 and erk1/2 in LM8 cells. Phosphorylation of (C) JNK, c-Jun and Akt and (D) p38 and erk1/2 in Dunn cells. FBS, fetal bovine serum.

Binding of CXCL12 to its cognate receptor CXCR4 is thought to activate several downstream pathways that regulate cell chemotaxis, survival, proliferation and migration (10). However, little is known regarding signaling pathways modulated by interactions of CXCL12 and CXCR4 in OS. To further identify specific downstream pathways by which CXCL12 and AMD3100 modulate OS behavior, including survival and metastasis, we performed western blotting to detect the phosphorylation levels of JNK, c-Jun, Akt, p38 and Erk1/2 in variously treated LM8 and Dunn cell groups. In LM8 cells, CXCL12 induced the phosphorylation of Akt, JNK and c-Jun, which was dose-dependently attenuated by AMD3100, but not the phosphorylation of p38 or erk1/2 (Fig. 5A and B). In the Dunn cells, none of these signaling pathway proteins were phosphorylated (Fig. 5C and D). These results indicated that activation of Akt, JNK and c-Jun occurred through CXCL12 binding to CXCR4, an effect that was weakened by blocking CXCR4 with AMD3100.

### The anti-apoptotic effect of CXCL12 depends on the inactivation of cleaved PARP

**Figure 6:**
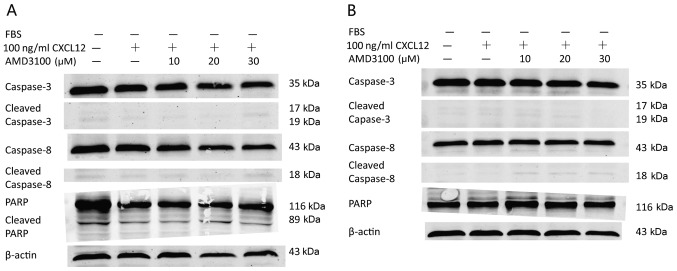
Anti-apoptotic effect of CXCL12 on CXCR4^+^ LM8 cells depends on inactivation of cleaved PARP. Expression of apoptosis-related protein caspase-3 and -8, and PARP were analyzed by western blotting. (A) Cleaved PARP was significantly downregulated in LM8 cells treated with 100 ng/ml CXCL12 compared with FBS-free controls. (B) Cleaved PARP is not detected in Dunn cells. FBS, fetal bovine serum.

As previously discussed, CXCL12 protects CXCR4^+^ LM8 cells from apoptosis induced by serum deprivation. However, the mechanism of this anti-apoptotic effect remains unclear. As the caspase family is crucial to tumor cell apoptosis (45), caspase-3 and -8, and PARP expression were detected by western blotting to investigate whether CXCL12 affected the regulation of caspase-dependent apoptosis. The presence of cleaved PARP, which ultimately leads to tumor cell apoptosis, was markedly decreased in the presence of CXCL12 in LM8 cells compared with the controls, although this result was not observed in Dunn cells (Fig. 6A and B). However, cleaved caspase-3 and -8 were not detected in LM8 and Dunn cells (Fig. 6A and B). These results showed that CXCL12 decreased apoptosis in serum-deprived CXCR4^+^ LM8 cells by decreasing cleaved PARP.

### AMD3100 inhibits primary tumor growth and lung metastasis in vivo

**Figure 7:**
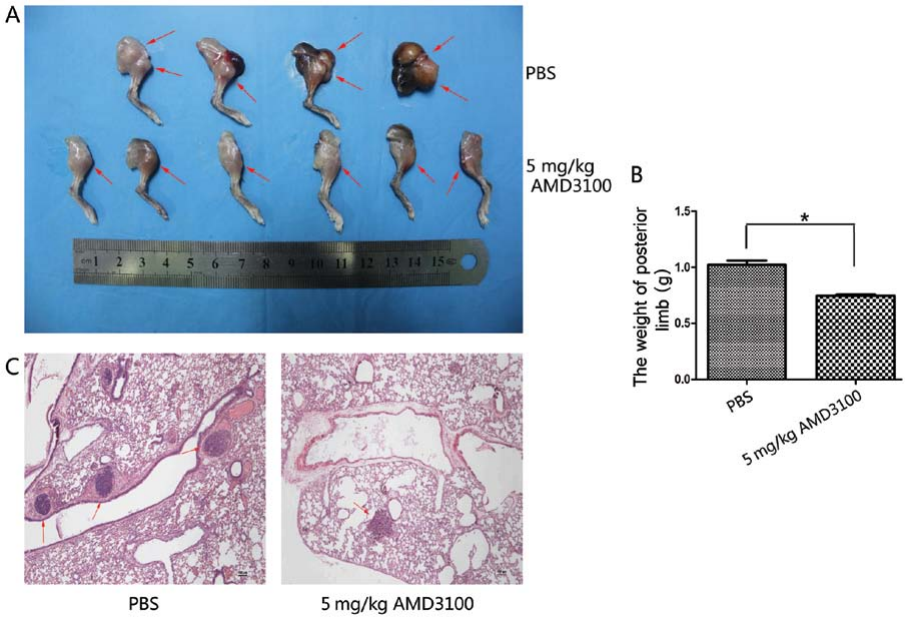
*In vivo* inhibitory effect of AMD3100 on primary and metastatic osteosarcoma (A). Tibial primary osteosarcoma tumors (red arrows) in C3H mice after treatment with 5 mg/kg AMD3100 or PBS (controls). Tumors in the AMD3100-treated group were significantly smaller than those of the controls. (B) Posterior limbs with primary tumor in the AMD3100-treated group were lighter than in the controls. (C) H&E stain of tumor lesions in lung tissues from the two groups (red arrows, ×40 magnification). FBS, fetal bovine serum.

To evaluate the tumor-inhibiting effect of AMD3100 *in vivo*, we established an orthotopic OS model by an intratibial injection of LM8 cells in mice, which were then injected with PBS alone (n=4, control group) or PBS with 5 mg/kg AMD3100 (n=6) every 2 days for 10 injections. The mice were then euthanized to observe primary tumor growth. Whereas the control group had large tumors, the tumors in AMD3100-treated group were not large enough to be observed with a naked eye (Fig. 7A). Posterior limbs with tumors resected from mice in the AMD3100-treated group were also lighter in weight than those of the control group (Fig. 7B). The H&E-stained lung tissues also showed tumor metastases to be notably smaller in the AMD3100-treated group than in the PBS control group (Fig. 7C).

## Discussion

Osteosarcoma (OS) has a markedly high risk of lung metastasis and poor survival. Accumulating evidence has confirmed involvement of the CXCL12-CXCR4 axis in the progression and metastasis of various types of cancer (18,19,34,46). To verify CXCR4 and/or CXCR7 participation in OS survival and metastasis, we first detected the expression of CXCR4 and CXCR7 in the murine LM8 and Dunn OS cell lines. LM8 was derived from Dunn using the Fidler method for generating metastatic clones of cancer cells. The metastatic potential of LM8 cells is higher than that of Dunn cells due to its higher expression of matrix metalloproteinases (MMPs)-2 and -9, vascular endothelial growth factor (VEGF) and β-catenin, which are crucial for metastasis (47). Consistent with that report, our western blotting results show that in LM8 cells, CXCR4 expression, which is widely considered to be an important biomarker for metastasis, is clearly higher than that in Dunn cells. Additionally, our FCM results show 4.1% of LM8 cells, but only 0.2% of Dunn cells, express cell-surface CXCR4. CXCR7, a novel decoy receptor of CXCL12, was identified in 2005 (48), and although its role in OS should also be taken into consideration, CXCR7 was not expressed in the LM8 or Dunn cells (Fig. 1A). Consistent with our observations, the study by Goguet-Surmenian *et al* (42) revealed that CXCR7 expression was undetectable in murine K7M2 and human SaOS-LM7 OS cells. Those authors indicated that CXCR7 was mainly expressed in tumor-associated blood vessels and rarely on tumor cells. CXCR7 was also not detected in human 143B OS cells by semi-quantitative RT-PCR and FACS analysis as reported by Brennecke *et al* (43). However, MG-63 and U-2OS OS cells, both expressing CXCR7, were utilized by Zhang *et al* to evaluate the role of CXCR7 in OS (44). As shown by our results, CXCR7 was not expressed in LM8 or Dunn cells, suggesting that CXCR4-CXCR7 crosstalk is not a factor when their ligand CXCL12 binds to LM8 or Dunn cells. In other words, only the CXCL12-CXCR4 axis affects progression and metastasis in these cells.

Previous studies have focused on the role of CXCR4 in OS metastasis (20–22), whereas little attention has been paid to CXCR4-mediated survival and growth in OS. Berghuis *et al* (36) reported that CXCL12 induced proliferation of serum-starved CXCR4^+^ Ewing sarcoma cells, and this effect was disturbed by AMD3100 *in vitro*. Concordantly, our results also showed the viability of serum-starved CXCR4^+^ LM8 cells in the presence of CXCL12 for 7 days to increase by 87% compared with the control groups, whereas this CXCL12-induced increased proliferation effect was not observed in CXCR4^−^ Dunn cells (Fig. 2). Furthermore, we found that AMD3100 abolition of CXCL12-induced survival of LM8 cells is not dose-dependent, and treatment with AMD3100 alone does not alter LM8 cell survival. However, a study by Kim *et al* (49) revealed that CXCL12 did not affect the proliferation of CXCR4^+^ myeloma cells, and AMD3100 has dual effects, initially enhancing and subsequently inhibiting myeloma cell proliferation. One possible explanation for the opposite results may be that the observation time points we selected are different. To be specific, we evaluated long-term (7 days) effects of CXCL12 and AMD3100, whereas shorter-term (3 days) effects were assessed by Kim *et al* (49).

Another important finding in the present study is that CXCL12 protects CXCR4^+^ LM8 cells from serum-deprivation-induced apoptosis by inactivating cleaved PARP. Activation of caspase family proteins can irreversibly initiate protein degradation and apoptosis (50,51). Caspase-3 is considered to be of great importance among the caspases since its activated form cleaves PARP, which ultimately results in the apoptosis of tumor cells (45,50). The percentage of apoptotic LM8 cells treated with CXCL12 decreased by 0.60-fold to 33.2% compared with the controls (Fig. 3). To determine the anti-apoptosis mechanism of CXCL12, we used western blotting to detect the expression of caspase-3 and -8, and PARP, which play important roles in apoptosis. Although cleaved caspase-3 and -8 were not expressed in LM8 or Dunn, cleaved PARP in CXCL12-treated LM8 cells was significantly less than in the control group.

We also investigated the role of CXCR4 in the migration of OS cells. Previous studies have shown CXCL12-CXCR4 interaction to mediate OS migration and invasiveness and effects can be abrogated by the CXCR4 inhibitor (20,22). Consistent with these findings, our results also indicate that the migration of CXCR4^+^ LM8 cells increases in the presence of CXCL12 *in vitro*, although pretreatment with AMD3100 impairs the migration-increasing effect induced by CXCL12. However, CXCL12 also induces the migration of CXCR4^−^ Dunn cells. It is likely that other undefined receptors in Dunn cells also contribute to migration in response to CXCL12. Further investigation is, however, required to confirm this hypothesis.

More importantly, binding of CXCL12 to CXCR4 stimulates the activation of downstream pathways that regulate cell chemotaxis, survival, proliferation and migration. Phosphoinositide 3-kinase (PI3K) and mitogen-activated protein kinase (MAPK) pathways have been found to critically affect tumor cell survival and migration, and are important downstream pathways involved in CXCL12/CXCR4 interactions, as summarized in our previous study (10). PI3K activation results in Akt phosphorylation, which induces the activation of nuclear factor-κB (NF-κB) transcription factors. MAPK pathways, which include extracellular signal-regulated kinase (ERK)-1/2, c-Jun N-terminal kinase (JNK) and p38, can also stimulate NF-κB expression (10). Chinni *et al* (52) demonstrated that CXCL12 induced MMP-9 expression in prostate cancer cells by activating PI3K-Akt-NF-κΒ and MEK pathways. Leelawat *et al* (53) suggested that the binding of CXCL12 to CXCR4 induced cholangiocarcinoma cell invasiveness by triggering the ERK1/2 and PI3K signaling pathways. To the best of our knowledge, this is the first study to identify specific downstream pathways, which we deem novel, regulated by CXCL12/CXCR4 interaction in the survival and metastasis of OS. Based on our results that CXCL12 stimulates the phosphorylation of Akt, JNK and c-Jun, which were attenuated by AMD3100 in LM8 cells, we conclude that CXCL12 induces the CXCR4-mediated survival and metastasis of OS by activating Akt and JNK pathways. Of note, this survival and metastasis-enhancing effect can be abolished by AMD3100 via the suppression of Akt and JNK pathways.

To the best of our knowledge, few studies have focused on the role of AMD3100 in inhibiting tumor growth and metastasis *in vivo*. Gros *et al* (35) reported that primary tumor growth and metastasis in a mouse model of esophageal cancer treated with 5 mg/kg AMD3100 by intraperitoneal injection were significantly reduced. Sun *et al* (7) also indicated that treatment with 1.25 mg/kg AMD3100 intraperitoneally twice daily for 4 weeks inhibited chondrosarcoma growth and metastasis. By contrast, Domanska *et al* (29) showed that treatment with 3.5 mg/kg AMD3100 alone for 5 weeks did not affect prostate tumor growth *in vivo* but sensitized prostate cancer to docetaxel chemotherapy. Despite findings of the AMD3100 effect on other tumors, its *in vivo* effect on OS growth remains to be determined.

To the best of our knowledge, the present study has shown, for the first time, the antitumor effect of AMD3100 in an OS C3H mouse model established by LM8 cells. Consistent with results with other types of cancer, we found that primary OS tumor growth and lung metastasis in C3H mice treated with 5 mg/kg AMD3100 by tail vein every 2 days for 10 injections were notably less than in the PBS control group.

In conclusion, a novel mechanism has been found to modulate survival and metastasis in OS. Due to a lack of CXCR7 expression, CXCL12 induces cell survival and migration of OS *in vitro* through CXCR4, which can be abrogated by AMD3100, a CXCR4-specific antagonist. The JNK and Akt pathways affect CXCR4-mediated OS progression and metastasis. The CXCL12-CXCR4 axis is therefore a potential therapeutic target in the treatment of OS.

## Abbreviations

OS: οsteosarcoma
CXCL12: chemokine 12
CXCR4: chemokine receptor 4
SDF-1: stromal cell-derived factor-1
HIF-1α: hypoxia-inducible factor-1α
CXCR7: chemokine receptor 7
DMEM-h: high-glucose Dulbecco’s modified eagle’s medium
FBS: fetal bovine serum
OD: optical density
FCM: flow cytometry
PE: phycoerythrin
FITC: fluorescein isothiocyanate
PI: propidium iodide
MMPs: matrix metalloproteinases
VEGF: vascular endothelial growth factor
PI3K: phosphoinositide 3-kinase
MAPK: mitogen-activated protein kinase
NF-κB: nuclear factor-κB
ERK: extracellular signal-regulated kinase
JNK: Jun N-terminal kinase
